# Glucocorticoid-induced adrenal insufficiency from a supplement containing undisclosed dexamethasone

**DOI:** 10.1210/jcemcr/luag277

**Published:** 2026-09-16

**Authors:** Hameeda Fatima, Lovepreet Johal, Ahmed Ibrahim, Gurinder Ghotra, Archana Reddy

**Affiliations:** Department of Internal Medicine, San Joaquin General Hospital, French Camp, CA 95231, USA; Department of Internal Medicine, San Joaquin General Hospital, French Camp, CA 95231, USA; Touro University California, College of Osteopathic Medicine, Vallejo, CA 94592, USA; Department of Internal Medicine, San Joaquin General Hospital, French Camp, CA 95231, USA; Department of Internal Medicine, San Joaquin General Hospital, French Camp, CA 95231, USA; Department of Internal Medicine, San Joaquin General Hospital, French Camp, CA 95231, USA

**Keywords:** glucocorticoid-induced adrenal insufficiency, adrenal insufficiency, ACTH, cortisol

## Abstract

Glucocorticoid-induced adrenal insufficiency (GIAI) is a very common cause of adrenal insufficiency, resulting from exogenous glucocorticoid exposure leading to suppression of the hypothalamic–pituitary–adrenal axis. While typically due to prescribed glucocorticoids, hidden ingredients in supplements may also contribute to this exposure. We describe a case of a 52-year-old male who presented with recurrent gastrointestinal symptoms of nausea and vomiting. Clinical features included persistent sinus tachycardia, hyponatremia, hypokalemia, and hypomagnesemia. The initial work-up was unrevealing; additional testing revealed a low random cortisol level, an inadequate response to cosyntropin stimulation, and an inappropriately normal adrenocorticotropic hormone level, confirming secondary adrenal insufficiency. Treatment with hydrocortisone resulted in improvement of the patient's clinical symptoms. Further history uncovered that the patient had abruptly discontinued a daily supplement named “Force Forever,” which, according to the US Food and Drug Administration (FDA), contains undisclosed ingredients, including diclofenac and dexamethasone. Although the FDA has issued a warning regarding this supplement, there have been no reported cases of GIAI from its use in the United States. This case emphasizes the importance of comprehensive history taking and considering adrenal insufficiency in the differential diagnosis for patients presenting with refractory, unexplained gastrointestinal symptoms like nausea and vomiting.

## Introduction

Adrenal insufficiency can result from impaired cortisol production from the adrenal cortex (primary adrenal insufficiency), or from the pituitary or hypothalamus (secondary adrenal insufficiency). In addition to tumors, trauma, and infection, secondary adrenal insufficiency can be caused by suppression of the hypothalamic–pituitary–adrenal (HPA) axis by exogenous glucocorticoid therapy. Glucocorticoid-induced adrenal insufficiency (GIAI) is a very common cause of secondary adrenal insufficiency due to widespread use and availability of glucocorticoids [1]. GIAI is a fairly new term and has been used by some authors interchangeably with adrenal suppression [2]. Cortisol, an endogenous glucocorticoid, is produced by the adrenal cortex in response to stimulation by adrenocorticotropic hormone (ACTH) secreted by the anterior pituitary. Release of ACTH is regulated by corticotropin-releasing hormone (CRH) from the hypothalamus. Together, the hypothalamus, pituitary gland, and adrenal gland make up the HPA axis, which regulates cortisol production through a controlled feedback mechanism. Exogenous glucocorticoids can suppress CRH and ACTH secretion through this feedback loop, leading to chronic suppression of the HPA axis and, over time, atrophy of the adrenal zona fasciculata [1, 3]. Therefore, abrupt withdrawal of exogenous glucocorticoids can cause adrenal insufficiency. The availability of nonprescription supplements containing undisclosed glucocorticoids, commonly marketed for pain control or performance enhancement, poses a risk of prolonged glucocorticoid use. Herein, we present a case of symptomatic GIAI secondary to unknown exogenous glucocorticoid consumption from supplements. This case report highlights the diagnostic challenge, along with the risks and complications, associated with use of such a supplement.

## Case presentation

A 52-year-old male, a construction worker by profession, with a past medical history of hypertension, hyperlipidemia, and a recent history of vertebral fracture, presented for evaluation of a syncopal episode while showering at home. He was found to be drowsy with slurred speech, which resolved spontaneously within 10 minutes. The patient endorsed a 1-week history of progressively worsening dizziness, now severe enough to require assistance with activities of daily living. In addition, he reported persistent nausea with postprandial vomiting of up to 3 episodes daily over the past 2 weeks. The patient denied any prior similar episodes, recent travel, infectious symptoms, or any substance use. Home medications included atorvastatin 40 mg daily, a combined tablet of lisinopril and hydrochlorothiazide (20/25 mg) daily, and a magnesium supplement.

## Diagnostic assessment

On arrival, the patient had sinus tachycardia (heart rate, 100-120 bpm), a blood pressure of 111/82 mm Hg, and normal oxygen saturation on room air. A complete blood count showed no anemia, leukocytosis, or eosinophilia. The comprehensive metabolic profile showed no hypoglycemia but revealed hyponatremia with a serum sodium concentration of 117 mEq/L (SI: 117 mmol/L) (reference range, 136-145 mEq/L [SI: 136-145 mmol/L]); hypokalemia with a serum potassium of 2.8 mEq/L (SI: 2.8 mmol/L) (reference range, 3.5-5.0 mEq/L [SI: 3.5-5.0 mmol/L]); hypomagnesemia with a serum magnesium of 1.4 mg/dL (SI: 0.58 mmol/L) (reference range, 1.7-2.2 mg/dL [SI: 0.70-0.91 mmol/L]); an acute kidney injury with a serum creatinine of 1.91 mg/dL (SI: 169 μmol/L) (reference range, 0.74-1.35 mg/dL [SI: 65-119 μmol/L]); and an estimated glomerular filtration rate of 37 mL/min/1.73 m^2^ (reference range, at least 60 mL/min/1.73 m^2^). The serum lactate concentration was elevated at 27.5 mg/dL (SI: 3.05 mmol/L) (reference range, 4.5-19.8 mg/dL [SI: 0.5-2.2 mmol/L]). Computed tomography (CT) head, computed tomography angiography head and neck, and CT chest/abdomen/pelvis did not reveal any acute abnormalities. An incidental 2-cm right adrenal nodule found on abdominal imaging was a myelolipoma. Magnetic resonance imaging of the brain was negative for posterior circulation stroke. The patient was treated with hypertonic saline for symptomatic hyponatremia, resulting in partial improvement of his symptoms. His presentation at this time was attributed to acute symptomatic hyponatremia and hypokalemia secondary to hydrochlorothiazide use, poor oral intake, and persistent vomiting. The patient was discharged after improvement in symptoms and normalization of electrolyte derangements.

The patient presented again 5 days later for persistent nausea, vomiting, abdominal pain, dizziness, and palpitations. On this admission, esophagogastroduodenoscopy (EGD), performed due to concern for persistent abdominal symptoms, was grossly normal; however, gastric biopsies were positive for *Helicobacter pylori*. On morning laboratory evaluation, the patient was found to have persistent hyperphosphatemia (>5.0 mg/dL [SI: >1.6 mmol/L]; reference range, 2.5-4.5 mg/dL [SI: 0.81-1.45 mmol/L]), for which parathyroid hormone levels were obtained and found to be within the normal range (17 pg/mL [SI: 1.8 pmol/L]; reference range, 15-65 pg/mL [SI: 1.6-6.9 pmol/L]). Additional history from the patient's wife indicated that the patient had been fully functional until 6 weeks before his initial hospital presentation. After further detailed questioning, he disclosed chronic daily use of an unregulated supplement from Mexico, marketed as “Force Forever,” for chronic knee pain due to his physically demanding job. He had used the supplement for nearly 1 year and abruptly discontinued it 2 months before the onset of his symptoms. US Food and Drug Administration (FDA) laboratory analysis confirmed that the product contained dexamethasone and diclofenac, which were not listed on its label [4]; the FDA report did not quantify the dexamethasone content. Additional diagnostic studies showed a markedly low random serum cortisol concentration of 2.6 µg/dL (SI: 71.7 nmol/L) (reference range, 6.2-19.4 µg/dL [SI: 171-536 nmol/L]). Cosyntropin stimulation testing demonstrated an inadequate cortisol response. The baseline serum cortisol concentration at 4:00 Pm was 2.0 µg/dL (SI: 55.2 nmol/L) (reference range for samples obtained between 4:00 and 6:00 Pm, 1.8-13.6 µg/dL [SI: 49.7-375.2 nmol/L]), with an inappropriately normal baseline ACTH concentration of 18 pg/mL (SI: 3.96 pmol/L) (reference range, 7.2-63.3 pg/mL [SI: 1.6-13.9 pmol/L]). After cosyntropin administration, the serum cortisol concentration increased to 10.0 µg/dL (SI: 276.0 nmol/L) at 30 minutes and 11.9 µg/dL (SI: 328.4 nmol/L) at 60 minutes. Both values were below the laboratory-defined normal peak response of greater than 20.0 µg/dL (SI: >552 nmol/L), supporting secondary adrenal insufficiency. In a patient with prolonged use and abrupt discontinuation of a glucocorticoid-containing supplement, these findings were highly suggestive of GIAI.

## Treatment

Initial management after EGD and *H. pylori-*positive biopsies included starting quadruple therapy, without any improvement in symptoms. Nutritional and dietary consultation was obtained, and customized meal plans were given without significant benefit. Psychiatric evaluation was requested after staff observed the patient inducing vomiting to relieve his intense nausea; the evaluation revealed no evidence of depression, an eating disorder, or another psychiatric diagnosis.

When clinical suspicion for GIAI became very high, additional testing, such as cosyntropin stimulation, was performed. Pending results, the patient was started on hydrocortisone therapy (15 mg in the morning and 5 mg in the evening) with drastic improvement in the patient's symptoms. Results of the cosyntropin stimulation test, available a few days later, confirmed secondary adrenal insufficiency likely due to exogenous use of glucocorticoids.

## Outcome and follow-up

Hydrocortisone replacement resulted in a remarkable improvement of his nausea and vomiting, with increased oral intake. He was discharged home shortly thereafter in stable condition with endocrinology follow-up arranged.

## Discussion

This case is an example of GIAI resulting from abrupt discontinuation after extended use of a supplement called “Force Forever,” found to contain undisclosed dexamethasone and diclofenac. Exogenous glucocorticoids suppress the HPA axis through feedback at both the hypothalamus and pituitary and can lead to adrenal atrophy and impaired endogenous cortisol production. In this patient, a low baseline serum cortisol concentration of 2.0 µg/dL (SI: 55.2 nmol/L), an inadequate peak response of 11.9 µg/dL (SI: 328.4 nmol/L) after cosyntropin stimulation, and an inappropriately normal ACTH concentration of 18 pg/mL (SI: 3.96 pmol/L) were consistent with secondary adrenal insufficiency. In patients with chronic glucocorticoid exposure, withdrawal symptoms of myalgias, fatigue, weakness, nausea, and sleep disturbance can occur due to increased inflammatory cytokines after abrupt glucocorticoid cessation [5].

Long-term suppression of CRH and ACTH following abrupt discontinuation or inadequate tapering of glucocorticoids can result in adrenal insufficiency and potentially adrenal crisis. Prolonged suppression of proopiomelanocortin-related peptides has been proposed to lead to myalgia, arthralgia, fever, and headache. Depressed central noradrenergic and dopaminergic systems contribute to nonspecific withdrawal symptoms, including anorexia, nausea, vomiting, and weight loss. Loss of the suppressive effect of glucocorticoids on calcium and phosphate metabolism can cause hypercalcemia and hyperphosphatemia; in our patient, persistent hyperphosphatemia was specifically observed [2, 6].

This case posed challenges that led to delayed recognition of adrenal insufficiency. Despite an adequate history from the patient, further collateral history from the patient's wife regarding supplement use prior to symptom onset was necessary. Patients may use products containing undisclosed glucocorticoids for prolonged periods without realizing the metabolic effects of glucocorticoids or the risks of abrupt discontinuation. Failure of products to specify ingredients, such as glucocorticoids, increases the risk of adrenal insufficiency and, in severe cases, adrenal crisis [5].

## Learning points

- GIAI can result from unregulated use of supplements containing undisclosed glucocorticoids.
- Abrupt cessation of exogenous glucocorticoids, whether prescribed or taken unknowingly, can cause adrenal insufficiency secondary to HPA axis suppression.
- Collateral history is an important diagnostic tool when patients may present with vague or nonspecific symptoms.
- A low random cortisol concentration with an inadequate response to cosyntropin stimulation in the setting of a normal ACTH level points to GIAI rather than primary adrenal disease.
- Unregulated or imported supplements carry significant pharmacological risk, and FDA safety warnings should be provided to those who use such products, given the potential for undisclosed active drug ingredients.

## Contributors

All authors made individual contributions to authorship. H.F., G.G., A.R., and A.I. were involved in the diagnosis and management of this patient. L.J. was involved in patient care as part of the clinical team. H.F. and A.R. critically revised the manuscript for important clinical content. All authors reviewed and approved the final draft.

## Data Availability

Original data generated and analyzed during this study are included in this published article.
